# Quality Improvement Interventions for Nutritional Assessment among Pregnant Mothers in Northeastern Uganda

**DOI:** 10.1155/2017/8036535

**Published:** 2017-05-30

**Authors:** Jonathan Izudi, Calvin Epidu, Andrew Katawera, Adeodata Kekitiinwa

**Affiliations:** ^1^Baylor College of Medicine, Children's Foundation-Uganda, Box 72052, Kampala, Uganda; ^2^Mulago National Referral Hospital, Block 5 Clock Tower, Kampala, Uganda

## Abstract

**Introduction:**

Assessment of pregnant mothers for nutritional status is a neglected intervention. In Kaabong Hospital, nutritional status of pregnant mothers was not assessed during antenatal care (ANC) visits. A quality improvement (QI) project was initiated to increase nutritional assessment using midupper arm circumference (MUAC) among pregnant mothers during ANC visits from 0 to 90% between April and September 2015.

**Method:**

Baylor-Uganda formed ANC Work Improvement Team (WIT) that reviewed ANC register, identified gaps in quality of care, analyzed root causes using cause-effect diagram, developed solutions, and tested and implemented the solution using Plan-Do-Study-Act cycles. Planned and tested changes included the provision of anthropometric tools, integrated ANC register, and data use.

**Result:**

In April 2015 (baseline), none (0/235) of the pregnant women were assessed for nutritional status using MUAC. Following QI interventions, nutritional assessment improved to 79% (200/252) in May 2015 and to 100% (241/241) in June 2015. The 100% performance was sustained until August 2016. Overall, 39 cases of malnutrition—1 (2.6%) severe (MUAC < 19.0 cm) and 38 (97.4%) moderate acute malnutrition (MUAC 19–22.0 cm)—were identified and linked to nutritional rehabilitation program.

**Conclusion:**

QI interventions are critical in achieving high rates of nutritional status assessment and identifying malnourished pregnant women during ANC visits.

## 1. Problem

Worldwide, malnutrition is a leading cause of morbidity and mortality. Of the global population, two billion people suffer from various forms of malnutrition and 2.6 million childhood deaths occur annually [1]. The harmful effects of malnutrition are more pronounced in pregnant and breast-feeding mothers as well as children below 3 years. Improving nutritional assessment in pregnancy is critical because malnutrition increases the risk of poor pregnancy outcomes such as low-birth weight (less than 2500 grams), premature delivery, and intrauterine fetal growth restriction [2]. For instance, severe anemia (hemoglobin level less than 5.0 g/dl) is linked to increased maternal mortality at delivery time [3]. This implies that maternal nutritional health status and pregnancy outcomes are interdependent [4, 5]. Indeed evidence indicates that moderate protein-calorie malnutrition during pregnancy leads to lower placental weight and this mechanism explains low-birth-weight babies [5]. Similarly, malnutrition in pregnancy leads to lower birth weight [4, 5], congenital malformations [4], and increased maternal and perinatal morbidity and mortality [4]. Therefore routine anthropometric measures such as body mass index (BMI) and a composite of weight in kilograms and height in meters-squared or midupper midarm circumference (MUAC) must be a key component of health service delivery.

However in Kaabong Hospital, Northeast Uganda, a substantial number of pregnant mothers that attend antenatal care (ANC) were not assessed by healthcare providers for nutritional status using MUAC.

This is contrary to the recommendations of Uganda's Ministry of Health requiring all pregnant mothers to undergo nutritional assessment at each ANC visit using MUAC measurements. The absence of nutritional assessment implies that routine nutritional counseling and dietary information provided to pregnant mothers are unguided. Secondly, numerous cases of malnourished pregnant mothers are missed to be diagnosed and timely interventions to improve maternal and newborn health and survival are not implemented. A quality improvement (QI) project was hence started at the ANC Clinic of Kaabong General Hospital. The objective was to increase nutritional assessment among pregnant mothers using MUAC from zero percent in April 2015 to 80% in November 2015. In this study, the parameter for nutritional assessment is MUAC, a measure of muscle mass [6].

## 2. Background

Until recently, Uganda focused mainly on the quantity of healthcare at the expense of quality [7, 8]. However, Uganda has now embarked on QI so as to close the gap between current and expected performance levels using appropriate solutions as defined by standards. Uganda's Ministry of Health launched its first QI strategic framework in 2010 and the launch was followed by the establishment of QI committees at various levels (national, regional, district, health subdistrict, hospitals and their departments, and health centers levels) [8]. The ultimate goal of the QI framework is the provision of high quality health services and the attainment of good quality of life and well-being at all levels of healthcare delivery in Uganda [7, 8].

Quality of healthcare is critical because it mediates the relationship between the six WHO (World Health Organization) building blocks of health systems strengthening (service delivery, health work force, health information, health financing, leadership, and medical products, vaccines, and technologies) and health outcomes (effectiveness, efficiency, responsiveness, and social and financial risk protection) [8].

Kaabong Hospital is owned by the Government of the Republic of Uganda and is located in Kaabong district, Karamoja region, Northeastern Uganda. Elsewhere [9], we described the sociodemographic, epidemiological, topographic, and health performance indicators of Karamoja region. Kaabong Hospital has a departmental QI team known as Work Improvement Team (WIT) formed by Baylor College of Medicine Children's Foundation (in short Baylor-Uganda) in 2015. The role of the WIT is to identify quality of care gaps and to devise strategies for addressing such gaps using a QI approach.

At the ANC Clinic of Kaabong Hospital, we reviewed three months of ANC data (January–March, 2015) in April 2015 and found that all pregnant women that attended ANC were not assessed for nutritional status. Health workers mainly measured weight without height or measured height without weight and MUAC was completely not measured. This was contrary to Ministry of Health-Uganda requirements of assessing all (100%) pregnant mothers for nutritional status at every ANC visit using MUAC. Moreover, nutritional guidelines require that MUAC measures must be performed at every ANC visit so as to assess nutritional status among pregnant mothers.

## 3. Baseline Measurement

To assess gaps in quality of care offered to pregnant mothers during ANC visits, we conducted a QI-oriented technical support supervision in the ANC unit of Kaabong Hospital in April 2015. We reviewed the ANC register and summarized its data into frequencies and percentages. We found none of the pregnant mothers that attended ANC in the previous months had weight and height recorded in the same ANC visit, none had MUAC measurement as well, 30% had blood pressure (BP) measurements, and 10% had their hemoglobin level estimated. Equally, there were no measures in place to ensure all pregnant mothers receive nutritional assessment during ANC visits. We then shared the findings with the ANC WIT that prioritized nutritional assessment using MUAC for improvement through consensus.

## 4. Design

We held a 1-hour Continuous Medical Education (CME) session on basic QI concepts (introduction to QI, dimensions of quality, quality grid, steps and principles of QI, data use, and QI tools used for problem identification and analysis among others). The purpose of the CME session was to build staff capacity and to refresh those previously trained in QI. During the CME session, illustrations were made using results from the ANC unit. At the end of the CME session, a QI project was started and the objective was to increase nutritional assessment among pregnant mothers using MUAC during ANC visits.

## 5. Strategy

This QI intervention followed four steps: first, the identification of gaps in nutritional assessment (problem identification and prioritization phase) using the ANC register; secondly, the identification of root causes of prioritized gap (problem analysis phase); thirdly, the development of simple, appropriate, and evidenced-oriented solutions (phase of development of improvement changes/solutions), and, fourthly, the testing and implementation of solutions (implementation phase) [10].

**Step 1 (identification of gaps via ANC data review).**

Data on nutritional indices (weight, height, and MUAC), blood pressure (BP), and hemoglobin level estimates were abstracted for all pregnant mothers that attended ANC between January and March 2015 in April 2015. The percentage of nutritional assessment using MUAC, BP, and hemoglobin level estimations was zero percent (0/235), 30% (70/235), and 10% (24/235), respectively. Zero percent nutritional assessment using MUAC was prioritized for QI through consensus.

**Step 2 (analysis of nutritional quality of care gap).**

**Figure 1:**
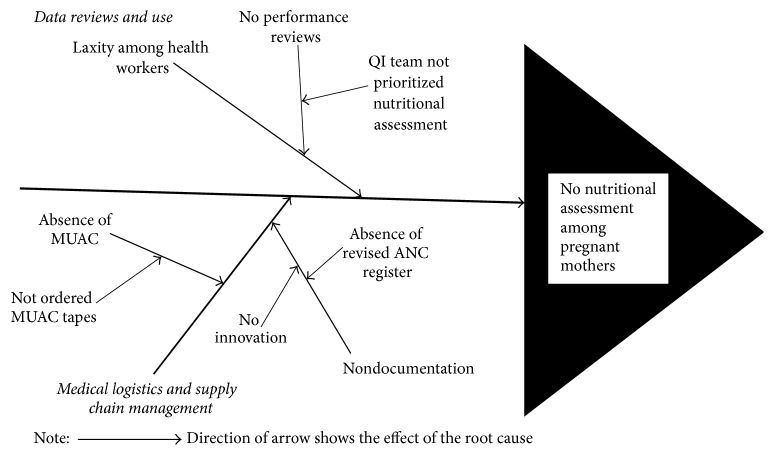
Fishbone tool for analysis of the root cause of low nutritional assessment using midupper arm circumference among pregnant mothers attending ANC Clinic, Kaabong Hospital.

The fishbone tool was used to analyze the root causes of zero percent nutritional assessment using MUAC among pregnant mothers during ANC visits [11]. The Why-Why-Why-Why-Why approach, a method of identifying root causes of the gap by asking the question “Why are pregnant women not assessed for nutritional status using MUAC during ANC visits?” to generate an answer that was subjected to further questioning. The process was repeated for each identified root cause/answer until no more answers/causes were identified. This approach ensured an exhaustive identification of underlying root causes of zero percent nutritional assessment [11]. The root causes were then categorized into medical logistics and supplies chain management and data reviews and utilization (Figure 1). In particular, the causes of weaknesses in logistics and supplies chain management included the lack of MUAC tapes and height boards because they were not ordered from the National Medical Stores of the Ministry of Health-Uganda. In relation to data reviews and data use, lack of priority given to nutritional assessment among pregnant mothers by ANC staff, lack of routine nutrition data reviews by the WIT and, laxity among ANC staff to conduct nutritional assessment were identified as principal root causes. Lastly, no documentation of nutritional parameters in the ANC register was due to lack of a column for recording (Figure 1).

**Step 3 (development and prioritization of improvement changes).**

**Figure 2:**
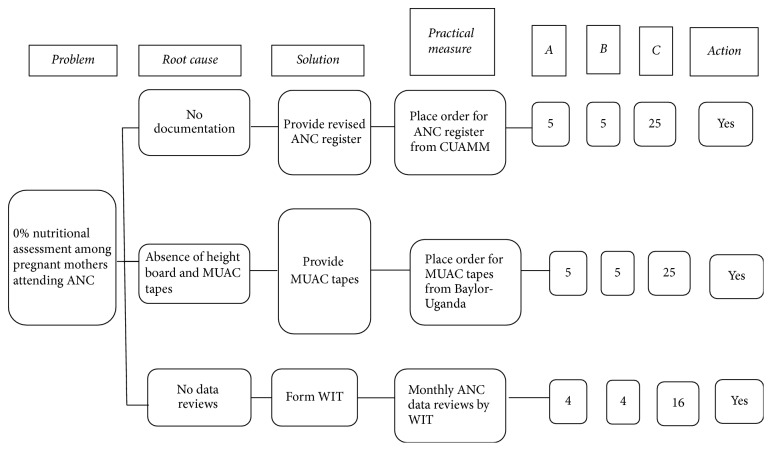
Countermeasures matrix diagram showing prioritization of quality improvement changes for root causes of nonnutritional assessment among pregnant mothers at ANC Clinic, Kaabong Hospital.* Note*. *A*: effectiveness score; *B*: feasibility score; *C*: product of *A* and *B*; action: Yes = accepted for quality improvement and No = rejected for quality improvement.

Based on identified root causes, we developed and prioritized three QI solutions (improvement changes) using countermeasures matrix (Figure 2). In the countermeasures matrix, the more feasible and effective the solution, the higher the ranking score (the scores ranged from 1 to 5) and vice versa. The overall score was a product of the feasibility and effectiveness ranking scores [12]. Placing an emergency order for a revised ANC register from an implementing partner (Doctors with Africa-CUAMM) was regarded feasible and effective with an overall score of 25. Placing an order for color-coded MUAC tapes from Baylor-Uganda was equally considered feasible and effective and scored 25. Lastly, starting monthly ANC nutritional data reviews and using the results to improve nutritional assessment scored 16 (Figure 2).

**Step 4 (implementation of improvement changes).**

We used the Plan-Do-Study-Act (PDSA) cycle, a series of steps for improving processes [13, 14], to test and implement improvement changes [15, 16], to plan, execute, observe and determine modifications needed during the implementation of improvement changes [13]. The PDSA Cycle ensured easy implementation, monitoring and evaluation, and modification of improvement changes [17]. During implementation, QI changes that never yielded positive results on were dropped or modified. In certain instances, new changes were developed.

*Documentation, Monitoring, and Evaluation of QI Process*. The QI documentation journal was used to track and report this intervention. The QI documentation journal had both start and end dates of the QI project, an objective (already stated in the design section), indicator, problem statement, planned and tested changes, and a line graph with annotations and lessons learnt. We measured height to two decimal places using standard height meters with the participant standing upright and barefoot on a flat surface at every ANC visit. Weight was measured on every ANC visit using calibrated scales with the participant wearing the same light cloth. All nutritional assessments and recordings were performed by a trained ANC Triage Midwife. The QI indicator was the proportion of pregnant mothers that received nutritional assessment using MUAC measurement only at each ANC visit. The numerator was the number of pregnant mothers assessed for nutritional status using MUAC at every ANC Clinic visit and the denominator was the total number of pregnant mothers that attended ANC on a particular day.

To obtain the total number of pregnant women whose nutritional status was assessed in a week, the daily number of pregnant women with a record of nutritional status assessment using MUAC was added. The ANC-QI Focal Person was responsible for summarizing the nutritional data, updating the QI documentation journal and giving a feedback on the progress of QI during monthly QI meetings.

## 6. Results

**Figure 3:**
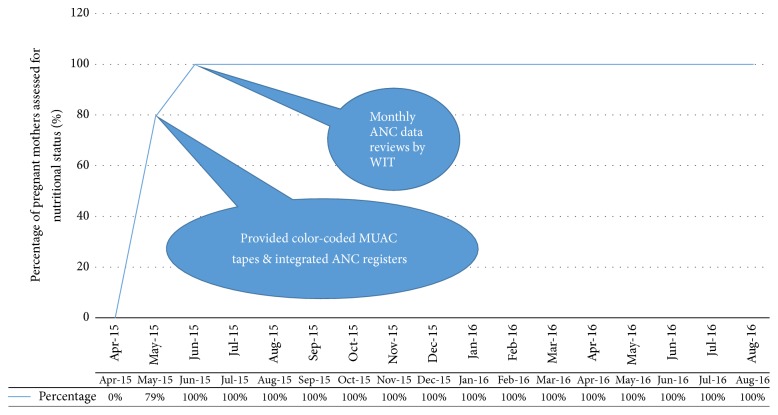
Line graph showing percentage of pregnant mothers whose nutritional status was assessed using midupper arm circumference during ANC visits, Kaabong Hospital.

The assessment of all pregnant mothers for nutritional status using MUAC increased from 0% (0/235) in April 2015 (baseline) to 79% (200/252) in May 2015 (Figure 3). This was attributed to the acquisition of color-coded MUAC tapes from Baylor-Uganda and revised ANC register from CUAMM (PDSA Cycle-1). There was another rise in nutritional assessment from 79% (200/252) in May 2015 to 100% (241/241) in June 2015 when the ANC WIT started monthly ANC data reviews to identify gaps (PDSA Cycle-2). Subsequently, the 100% performance was maintained until August 2016 (Supplementary Material S1, available online at https://doi.org/10.1155/2017/8036535).

During this QI intervention, 39 (MUAC < 22.0 cm) cases of malnourished pregnant mothers were identified. Of the 39 malnourished cases, one (2.6%) had severe acute malnutrition (MUAC < 19.0 cm) and was linked to the therapeutic feeding program within the hospital. 38 (97.4%) had moderate acute malnutrition (MUAC 19–22 cm) and were linked to the supplementary feeding program within the same hospital.

## 7. Lessons Learnt

This paper highlighted evidence-based interventions for improving nutritional assessment among pregnant mothers attending ANC in a remote, rural, public health facility in Northeast Uganda.

The provision of color-coded MUAC tapes and revised ANC register led to dramatic increase in nutritional assessment among pregnant mothers attending ANC. These QI changes demonstrate the importance of strengthening logistics and supplies chain management as critical catalysts/pathways in enhancing health systems performance. Our QI interventions suggest that improving logistics and supply chain management is indispensable in better health systems performance and high quality patient care. According to WHO, a well-functioning health system must ensure equitable access to essential medical products, vaccines and technologies of assured quality, safety, efficacy, and cost-effectiveness and their scientific soundness as well as cost-effective use [18]. Initially, our setting conflicted with this recommendation because the ANC unit lacked the essential supplies for nutritional assessment (MUAC tapes) in pregnant mothers hence the poor nutritional performance indicator.

We found the routine ANC data reviews ensured sustained performance in nutritional assessment among pregnant mothers. This demonstrates the importance of effective data management and utilization in health systems. In line with earlier recommendations [19], we found monthly data reviews were essential in identifying pitfalls in nutritional assessment, taking prompt corrective actions, and tracking progress.

However, much emphasis should be on data quality as poor quality data results into underutilization of data and inappropriate decisions. Similar to earlier report in Zambia [20], nutritional data was not used to improve maternal health services before this intervention due to poor quality data. In order to effectively address health systems challenges and improve the quality of service delivery, high quality data must be collected and analyzed to aid decision making [20]. Because nutritional data was collected systematically, accurately, and on purpose, several malnourished pregnant mothers that otherwise would have been missed were identified and linked to nutritional rehabilitation program.

## 8. Conclusion

Our results underscore the immense importance of nutritional assessment among pregnant mothers. In particular, it highlighted the importance of integrating QI in nutritional assessment and counseling and support at all health service delivery points. QI interventions should therefore be integrated at all service delivery points within the healthcare system. Health facilities with similar operational challenges may apply theses interventions.

## 9. Limitation

This paper is the first in Uganda to report improvement of nutritional assessment among pregnant mothers through a rigorously conducted QI project. Our paper underscored the need to focus on all the six WHO health systems building blocks in order to remarkably improve quality of healthcare. Secondly, it demonstrated the importance of QI in healthcare and adds new knowledge to literature.

However, the interventions did not focus on all the six WHO health systems building blocks that impact on quality of health services. Another pitfall is the lack of temporal relationship between documentation of nutritional status in the ANC register and actual nutritional status assessment (as lack of information on nutritional assessment in the ANC register may suggest both situations). In addition, the presence of the study itself might have contributed to improvement in nutritional status assessment as well as documentation as behaviors of health workers and knowledge might have changed over time. The low number of pregnant mothers that attend ANC visits on monthly basis is another limitation as a small change in the numerator produces large difference in proportion of nutritional assessment. Lastly, all nutritional assessments were performed cross-sectionally rather than longitudinally. The results of this paper should hence be interpreted in light of these limitations.

## Supplementary Material

Table showing pregnant mothers assessed for nutritional status at Kaabong Hospital ANC Clinic.

## Abbreviations

ANC: Antenatal care
CME: Continuous Medical Education
CUAMM: Doctors with Africa
MUAC: Midupper Arm Circumference
PDSA: Plan-Do-Study-Act
QI: Quality improvement
WHO: World Health Organization
WIT: Work Improvement Team.
