# Supplementary material for: Quality Improvement Interventions for Nutritional Assessment among Pregnant Mothers in Northeastern Uganda
Source: Biomed Res Int. 2017 May 30;2017:8036535. doi: 10.1155/2017/8036535 (PMC5468564; doi:10.1155/2017/8036535)
Supplement: Supplementary file 1 — Table showing pregnant mothers assessed for nutritional status at Kaabong Hospital ANC Clinic. [file 8036535.f1.pdf]

Supplementary material S1: Pregnant mothers eligible for nutritional assessment at ANC clinic, Kaabong Hospital

**Year**	**2015**									**2016**					
Months	Apr	May	Jun	Jul	Aug	Sep	Oct	Nov	Dec	Jan	Feb	Mar	Apr	May	Jun
Numerator	0	200	241	282	266	252	286	215	218	276	222	237	205	238	246
Denominator	235	252	241	282	266	252	286	215	218	276	222	237	205	238	246
Percentage	0	79	100	100	100	100	100	100	100	100	100	100	100	100	100
